# Areca nut chewing and systemic inflammation: evidence of a common pathway for systemic diseases

**DOI:** 10.1186/1476-9255-9-22

**Published:** 2012-06-07

**Authors:** Kashif Shafique, Saira Saeed Mirza, Priya Vart, Abdul Rauf Memon, Moin Islam Arain, Muhammad Farooq Tareen, Zia Ul Haq

**Affiliations:** 1 Institute of Health & Wellbeing, Public Health, 1-Lilybank Gardens, University of Glasgow, Glasgow, G12 8RZ, UK; 2 Department of Physiology, Institute of Basic Medical Sciences, Dow University of Health Sciences, Karachi, 74000, Pakistan; 3 Department of Health Sciences, University Medical Centre Groningen, Groningen, Netherlands; 4 Department of Medicine, Civil Hospital Karachi, Karachi, 71000, Pakistan; 5 Department of Medicine, Isra Medical University, Hyderabad, Pakistan; 6 Department of Health, Government of Balochistan, Quetta, Pakistan

**Keywords:** Systemic inflammation, Areca nut, Cancers, Cardiovascular diseases

## Abstract

**Background:**

Areca nut, the seed of fruit of an oriental palm, known as *Areca catechu,* is commonly chewed in many countries*.* Diabetes, hypertension, cardiovascular diseases, oropharyngeal and oesophageal cancers have been associated with areca nut chewing and the mechanism by which areca nut chewing increases the risk of systemic diseases remains elusive. We hypothesize that systemic inflammation may be elevated among areca nut users, which is linked with many systemic diseases. Therefore, this present study was conducted to examine the systemic inflammation among areca nut chewers and healthy controls.

**Methods:**

This was an observational cross sectional study carried out on areca nut chewers and healthy individuals in Karachi, Pakistan. Participants were selected from a region of the city by invitation request sent from door to door. Information was collected regarding the socio-demographic profile and the pattern of use, and a blood sample was obtained to measure the level of C-reactive protein (CRP). We carried out multiple logistic regressions to investigate the association between socio-demographic profile, areca nut chewing and CRP levels.

**Results:**

We carried out final analysis on 1112 individuals of which 556 were areca nut chewers and 556 were the age, gender and area matched controls. Areca nut chewers had a significantly higher proportion of men (15.1%, n = 84) who had an elevated CRP (>10 mg/dl) as compared to controls (5.2%, n = 29). Multivariate analyses showed that areca nut chewers had significantly higher odds of an elevated CRP (OR = 3.23, 95% CI 2.08-5.02, p value <0.001) as compared to controls. Increase in amount of areca nut consumption had a significant dose–response relationship with systemic inflammation (p for trend <0.001). Further analysis revealed that areca nut chewers with tobacco additives were two times more likely to have an elevated CRP as compared to raw areca nut users. These associations remained unchanged after adjustments for age, BMI and years of full time education.

**Conclusions:**

Areca nut chewing has a significant association with systemic inflammation. Further work is required to confirm that systemic inflammation is the main pathway by which areca nut use increases the risk of systemic diseases.

## Background

Areca nut is the seed of the fruit of a tropical palm tree, *Areca Catechu*. This tree bears fruit throughout the year and areca nut is obtained from it, which is a basic ingredient of widely used chewing products [1]. Slices of areca are used fresh or dried and occasionally cured before use by boiling, baking or roasting. These nuts are chewed either in raw form or mixed with a variety of substances including slaked lime (aqueous calcium hydroxide paste), artificial sweeteners, spices such as cardamom, coconut, saffron and most significantly, these nuts are also mixed with some tobacco products [2,3].

Areca nut is considered to be the fourth most commonly used psychoactive substance after tobacco, alcohol and caffeine and more than 10% of the World’s population chew it regularly more commonly in Central, Southern and South-east Asian countries [4]. Interestingly, the number of areca chewers has remarkably increased in Australia, United Kingdom, Europe and USA especially among the immigrants settling in these countries from the developing world [1]. Prevalence of areca nut chewer varies in different regions of the world with a higher prevalence in developing countries and lower in the developed part of the world [1].

Substantial body of evidence now suggests that areca nut chewing is associated with benign and malignant diseases of the oral cavity. WHO International Agency for Research on Cancer Monograph Working Group (2009) highlighed that the evidence on areca nut and its assosciation with oral, pharyngeal and esophageal cancer is sufficient to establish a causal link [5]. Interestingly, hepatocellular carcinoma, for which hepatitis B and C infections are considered as major risk factors, has been found commonly among areca nut chewers who were free from hepatitis B/C infections [6]. There is also growing evidence that areca nut chewing is associated with many systemic diseases including obesity [7,8] diabetes mellitus [9,10], hypertension [10] and metabolic syndrome [11]. Furthermore, areca nut chewing also increases the risk of cardiovascular diseases and ultimately increases all-cause mortality independent of traditional risk factors of these conditions [12,13].

Although, evidence suggests that areca nut chewing increases the risk of many systemic diseases, the underlying biological mechanisms of these associations remain elusive and little has been known and published in this area. There is compelling evidence that the alkaloids of areca nut have a vital role in the initiation and progression of oral and pharyngeal lesions by initiating local inflammation but there is no evidence as such that systemic inflammation has some role in the development of systemic and metabolic diseases among areca nut chewers. We hypothesize that systemic inflammation could be a main pathway by which areca nut consumption increases the risks of systemic diseases. Therefore, the present study was conducted to examine the systemic inflammation among areca nut chewers in comparison to the general population.

## Methods

### Cohort selection

We included healthy individuals from population for this study who were invited to outdoor patients department of Civil Hospital Karachi, Pakistan. The details of areca nut chewers and their selection have been described elsewhere [14]. In brief, all willing healthy male individuals between the ages of 16 to 35 who visited the outdoor patient department during January 2009 to December 2010 in response to the invitation were included in this study. Apparently healthy men who were chewers of areca products (i.e. Areca nut only or Areca nut with tobacco additives) were included in this study as cases. We prepared a list of 50 areca nut products commonly available in the market, so that individuals can be classified in groups accurately. Individuals who visited the specially setup clinic in out patient department for participation in this study and had current illness or wanted to had a follow up of a previous illness were not included in this study. Individuals who refused to participate in the survey or were unable to understand Urdu (national language of Pakistan) were also excluded from this survey.

Information about the basic demography and substance use was collected by using a questionnaire. This was a 20-items questionnaire including the information about socio-demographic profile (including age, years of education, nature of job/study), pattern of substance use (frequency, number of years of usage, daily consumption, type of substance and family history of use). This questionnaire was assessed for comprehensiveness, relevance and clarity by three health professionals. Based on their comments and suggestions some items were modified while five items were excluded. This was then piloted on 50 participants. The performance of the questionnaire was assessed by validity and reliability analysis which showed a fairly high validity and reliability with a Cronbach’s alpha for the studied sample as 0.90.

Men who responded to the invitation, who never chewed areca products, smoked cigarettes or used any other addictive substance were included in this study as controls. Furthermore, potential participants from community were also approached by local political leaders and key informants. Information about this particular study was also conveyed in community in different social gatherings to increase the community participation in this study. Controls were selected based on age, gender and area matched to cases. Any individual who came to participate in the study and had a self-reported history of any acute (acute febrile illnesses i.e. upper and lower respiratory tract and gastro-intestinal infections) or chronic illness (e.g. hypertension, diabetes mellitus, arthritis, asthma, COPD) were excluded from this study. Participants were examined by a dentist at recruitment site and those who had periodontal diseases were also excluded from this study. An additional questionnaire including the dietary as well as lifestyle habbits was also filled by an interviewing physician or nurse. General physical examination was conducted for all participants including the anthropometric measurements following a standard procedure of removing the shoes for height and weight measurements. A single page information sheet was designed to convey the aims and objectives of the study to the participants and a verbal consent was obtained prior to employing the questionnaire. A blood sample was obtained to measure the level of circulating C-reactive protein and other haematological profile of the participants. All blood samples were collected in serum-separating tubes containing anticoagulant in it. These samples were then processed to a biochemistry laboratory where they were analysed for CRP using Enzyme-linked immunosorbent assay (ELISA). The Novex manufactured 96-well microplate reader and CRP human ELISA kits were used for this analysis. This study was approved by the hospital authorities and an independent ethical committee also reviewed and approved the study protocol.

### Sample size estimation

We estimated the sample size to measure 6% difference of dependency prevalence (assuming 20% prevalence of systemic inflammation among areca users) between groups at 0.05 significance level using two sided comparison and power of 80%. Sample size was computed for both the *X*^*2*^ using the Yates’ continuity correction and Fisher Exact test. A sample of 938 participants was the minimum number required to be accrued to perform this survey with an allocation ratio of 1.

### Data analysis

We used Stata software version 11 (StataCorp, College Station, TX, USA) to analyze the collected information. Participants were divided into two groups according to their substance consumption (“areca nut chewers” and “controls”). Age was categorised into five years age bands (age years 16–20,21-25,26-30 and 31–35) and education into three categories <10 years, 10–12 years and >12 years. Body mass index (BMI) was calculated from weight (in kg) divided by height (in metres) squared and categorised according to the World Health Organisation (WHO) Asian population specific classification in which BMI <18.5 is underweight, <23 is desirable, 23–27.5 is overweight and >27.5 is classified as obese [15]. In our sample only two individuals had BMI less than 18.5 so we combined the underweight category with desirable group. C-reactive protein was dichotomised; those who had a CRP less than or equal to 10 mg/dl and CRP greater than 10, a cut off which has been consistently used in many studies to assess the systemic inflammation [16,17]. We used multiple logistic regression adding different explanatory variables to assess the association between areca nut use and systemic inflammation. We also investigated the interaction between different variables included in the multivariate model.

## Results

A total of 1112 men were included in this study, 556 areca chewers and 556 healthy controls. Mean age of participants was 25.6 ± 5.6 years with no statistically significant difference between cases and controls (p value = 0.73). Mean BMI of the sample was 25.8 ± 4.0 kg/m^2^, no significant difference was observed between cases and controls (p value 0.17). Significant differences were observed in years of full time education between cases and controls; controls were significantly more likely to be less educated as compared to cases (p value 0.001). Baseline distribution of sample is summarized in Table 1.

**Table 1:** Baseline characteristics of areca nut chewers and controls

**Characteristics**	**Number (n)**	**Areca nut chewers, n (%)**	**Controls, n (%)**	***p* value**
**Study participants (n)**	1112	556	556	
**Age at screening, mean (SD)**	1112	25.6 (5.6)	25.7 (5.6)	0.734
**Age at screening (years)**				0.641
Age 16-20	203	98 (17.6)	105 (18.9)	
Age 21-25	425	211 (38.0)	214 (38.5)	
Age 26-30	237	127 (22.8)	110 (19.8)	
Age 31-35	247	120 (21.6)	127 (22.8)	
**Body Mass Index, mean (SD)**	1112	25.7 (4.0)	26.0 (4.2)	0.166
**Body Mass Index (kg/m2)**				0.492
Desirable < 23	317	162 (29.1)	155 (27.9)	
Overweight 23–27.5	473	242 (43.5)	231 (41.6)	
Obese > 27.5	322	152 (27.3)	170 (30.6)	
**Education (years)**				< 0.001
< 10 years	167	66 (11.9)	101 (18.2)	
10 - 12 years	497	278 (50.0)	219 (39.4)	
> 12 years	448	212 (38.1)	236 (42.5)	
**C - reactive protein (mg/dl), mean (SD)**	1112	6.1 (3.8)	3.5 (3.6)	< 0.001
**Inflammation**				< 0.001
Absent (CRP ≤ 10 mg/dl)	999	472 (84.9)	527 (94.8)	
Present (CRP > 10 mg/dl)	113	84 (15.1)	29 (5.2)	

The mean CRP level significantly differed (mean difference 2.59, p-value <0.01) between cases and controls with higher level among cases (6.11 ± 3.8) compared with controls (3.53 ± 3.6). The proportion of men who had an elevated level of CRP (CRP >10 mg/dl) also significantly differed between cases and controls, where 15.1% (n = 84) areca nut chewers had an elevated CRP compared to 5.2% (n = 29) controls. On logistic regression analysis, there was no significant difference in likelihood of elevated CRP between age, BMI and education categories. However, areca nut chewers had 3 times higher odds of having an elevated CRP as compared to controls (OR 3.23, 95% CI 2.08-5.02, p value < 0.001). This association remained significant and slightly strengthened after adjusting for age, BMI and years of education (Table 2).

**Table 2:** Systemic inflammation and role of areca nut chewing, a comparison with healthy controls

	Univariate analysis			Multivariate analysis		
	Odds ratio (95% CI)	Wald Chi2	p value	Odds ratio (95% CI)	Wald Chi2	p value
**Age at screening (years)**						
Age 16-20	1			1		
Age 21-25	0.83 (0.48 - 1.44)	−0.66	0.51	0.79 (0.44 - 1.40)	−0.81	0.42
Age 26-30	0.97 (0.53 - 1.78)	−0.10	0.92	0.89 (0.47 - 1.66)	−0.37	0.71
Age 31-35	1.01 (0.56 - 1.83)	0.03	0.98	0.99 (0.54 - 1.83)	−0.02	0.99
**BMI (kg/m2)**						
Desirable < 23	1			1		
Overweight 23–27.5	0.90 (0.56 - 1.45)	−0.41	0.68	0.91 (0.56 - 1.47)	−0.38	0.71
Obese > 27.5	1.05 (0.63 - 1.74)	0.19	0.85	1.11 (0.66 - 1.85)	0.39	0.7
**Education (years)**						
< 10 years	1			1		
10 - 12 years	1.18 (0.65 - 2.16)	0.55	0.58	0.94 (0.51 - 1.76)	−0.18	0.86
> 12 years	1.16 (0.63 - 2.14)	0.47	0.64	1.07 (0.57 - 2.01)	0.22	0.83
**Group**						
Control	1			1		
Areca nut chewers	3.23 (2.08 - 5.02)	5.23	< 0.001	3.30 (2.11 - 5.15)	5.26	< 0.001

Multivariate model included all the co-variates listed in the table.

**Figure 1:**
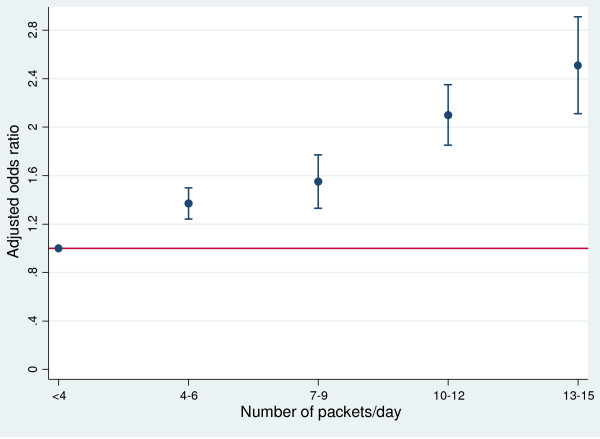
**The dose–response relationship between systemic inflammation and areca nut chewing.** Adjusted odds ratio calculated from a multivariate model adjusted for age, BMI and years of full time education.

The mean CRP level significantly differed (mean difference 1.89, p-value <0.01) between raw areca nut chewers and areca nut chewers with tobacco additives with higher level among areca nut chewers with tobacco additives (7.18 ± 3.95) compared with raw areca nut chewers (5.29 ± 3.44). The proportion of men with an elevated CRP significantly differed between raw areca nut chewers (12.1%, n = 38) and areca chewers with tobacco additives (19.0%, n = 46). On sub-group analysis, the controls were excluded from the analysis and likelihood of elevated CRP was compared between raw areca nut users and areca chewers with tobacco additives. Men who used areca nut with tobacco additives were two times more likely to have a higher CRP as compared to raw areca nut users (OR 1.98, 95% CI 1.16-3.31, p value 0.009) after adjusting for age, BMI and years of education (Table 3). Increase in amount of areca nut consumption also showed a significant (p for trend <0.01) dose–response relationship with systemic inflammation (Figure 1).

**Table 3:** Systemic inflammation and areca nut chewing with tobacco additives, a comparison with raw areca nut chewers

	Univariate analysis			Multivariate analysis		
	Odds ratio (95% CI)	Wald chi2	p value	Odds ratio (95% CI)	Wald chi2	p value
**Age at screening (years)**						
Age 16-20	1			1		
Age 21-25	0.83 (0.48 - 1.44)	−0.66	0.51	1.03 (0.51 - 2.11)	0.09	0.93
Age 26-30	0.97 (0.53 - 1.78)	−0.10	0.92	1.13 (0.52 - 2.43)	0.30	0.76
Age 31-35	1.01 (0.56 - 1.83)	0.03	0.98	0.57 (0.26 - 1.24)	−1.41	0.16
**Age at screening (continuous)**	0.98 (0.94 - 1.02)	−1.14	0.25	0.97 (0.93 - 1.01)	−1.28	0.20
**BMI (kg/m2)**						
Desirable < 23	1			1		
Overweight 23–27.5	0.90 (0.56 - 1.45)	−0.41	0.68	0.80 (0.45 - 1.43)	−0.75	0.46
Obese > 27.5	1.05 (0.63 - 1.74)	0.19	0.85	1.08 (0.59 - 1.98)	0.24	0.81
**Education (years)**						
< 10 years	1			1		
10 - 12 years	1.18 (0.65 - 2.16)	0.55	0.58	1.67 (0.73 - 3.82)	1.22	0.22
> 12 years	1.16 (0.63 - 2.14)	0.47	0.64	1.26 (0.55 - 2.87)	0.55	0.58
**Group**						
Raw areca nut chewers	1			1		
Areca with tobacco additives	1.70 (1.07 - 2.72)	2.24	0.03	1.98 (1.16 - 3.31)	2.61	0.009

Multivariate model included all the co-variates listed in the table.

**Figure 2:**
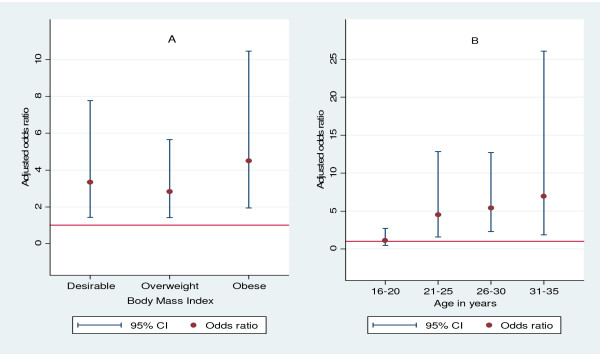
**Age and BMI specific risk of systemic inflammation among areca nut chewers as compared to controls.A** = BMI stratified analysis, **B** = Age-specific analysis. Adjusted odds ratio calculated from a multivariate model adjusted for age and years of full time education.

On interaction analysis, we did not find any significant interaction (p-value for interaction 0.33) between BMI and age of areca nut chewers in relation to systemic inflammation. However further age and BMI stratified analysis was carried out to control for any residual confounding. On age-specific analysis, areca nut chewers had significantly higher odds of having an elevated CRP as compared to controls except among subjects aged 16–20 years (Figure 1). On BMI stratified analyses, all areca nut chewers had significantly higher odds of having an elevated CRP with highest effect in the obese group (Figure 2).

## Discussion

The findings of the present study indicate that areca nut chewing is significantly associated with systemic inflammation as measured by CRP. The results suggest that areca nut chewing has a direct relationship with CRP independent of age, BMI and educational level. As expected, the users of areca nut with tobacco additives had even higher levels of CRP as compared to raw areca nut chewers. Moreover, areca nut chewing has a significant relationship with elevated CRP either measured as a continuous or categorical variable.

The major constituents of areca nut are carbohydrates, lipids, proteins, alkaloids and polyphenols[18]. There is preliminary evidence from *in vivo* data, that areca nut extracts augment the inflammatory response as well as the expression of tumor necrosis factor-alpha in mice models [19]. Extracts of both ripe areca nut and extracts of tender areca nut significantly enhanced the production of tumor necrosis factor-alpha and interleukin-1beta in peripheral blood mononuclear cells in a dose-dependent and time-dependent manner [20]. To the best of our knowledge, our findings provide the first evidence which shows the relationship of areca nut chewing and systemic inflammation measured by CRP in humans. The results of this study are biologically plausible, as areca nut consumption has been associated with the risk of developing diabetes mellitus, hypertension, cardiovascular diseases and cancers [8,11,13,21]. On the other hand, compelling evidence has shown that inflammation is a primary aetiological factor in development of hypertension [22,23], cardiovascular diseases [24,25] and cancers [26,27]. Taken together, areca nut might be increasing the risk of cardiovascular diseases and cancers by augmenting the systemic inflammatory response and elevating the levels of circulating inflammatory markers. Regular high consumption of areca nuts for longer duration may cause chronic inflammation, locally in oral cavity and systemically, which could lead to multiple diseases.

Our results may have been influenced by the tobacco components of areca nuts, but stratified analysis also showed the higher odds of systemic inflammation among raw areca nut chewers compared to healthy controls (data not shown). The confounding effect of age and obesity on systemic inflammation may have biased our results, although, the positive (harmful) association of areca nut chewing and systemic inflammation remained significant after adjustments for age and BMI, we also stratified the analysis based on age and BMI categories to remove the residual confounding due to these factors. The overall associations remain significant in stratified analysis except among the younger age group (age 16–20 years). The null association of areca nut chewing and systemic inflammation among younger participants could be explained by many factors including, less consumption of the nut or smaller duration since the beginning of the use. Furthermore, younger individuals tend to be physically active while participating in outdoor sports or leisure activities; exercise and physical activity has an inverse relationship with inflammation, reported by many studies [28,29]. So the harmful effect of areca nut chewing may actually be attenuated by some other unknown lifestyle factors resulting in a null finding in the younger age group, which we have reported.

The major strength of this cross sectional study was that it was population-based and we had a fairly large sample to examine associations between areca nut chewing and systemic inflammation. Although, our findings suggest that areca nut chewing has a strong relationship with systemic inflammation and hence may have a role in the development of systemic diseases at a later stage but causality can not be established in a cross-sectional study. The contribution of areca nut chewing in development of chronic diseases, should, therefore be assessed by larger prospective studies in relation to systemic inflammation.

This study has several limitations which need to be mentioned. As the participants were selected from one region of this city, so given the sample limitations one can not draw conclusions about the population level estimates from this study. We used only male individuals; therefore, it is difficult to assume that the prevalence of systemic inflammation would also be similar among the female population in this country. Indeed it may be even higher compared to males, as an earlier study from Taiwan suggested that the risk of hypertension was significantly higher among female areca nut chewers as compared to males [10]. Most of the individuals included in our study were from average or below average socio-economic class, so the results are not generalisable to all the social classes of this country.

### Public health implications

Areca nut use poses significant and avoidable morbidity and mortality. The present finding suggests a possible mechanism by which areca nut chewing is related with chronic diseases. Confirmation of causality between areca nut use and systemic inflammation and then further development of chronic disease would enhance the significance of primary prevention programs. Health education and awareness are perhaps the most crucial interventions required to be delivered so that the adverse effects of the substance can be appreciated by the community. Addressing a culturally sanctioned substance in terms of addiction and harm may meet with considerable resistance, especially if attention resulted in compromised supply or increased cost [30]. These interventions need to be focused on all age groups, particularly in early age school children to avoid future morbidity and mortality associated with this substance.

## Conclusions

Areca nut chewing was significantly associated with systemic inflammation. Systemic inflammation was significantly higher among men using areca nuts with tobacco additives. Further evidence is required to confirm the findings and its relationship with development of cardiovascular diseases and cancers.

## Competing interests

The authors declare that they have no competing interests.

## Authors’ contributions

SSM and KS conceived the idea and design of this study. SSM and MIA were involved in the drafting of questionnaires and collection of data. PV, KS, ZUH and MFT analysed the data and wrote the initial draft of this manuscript. All authors were involved in the interpretation of data and the critical revision of the manuscript for important intellectual content. All authors approved the final draft for publication.
